# Distribution of traditional Chinese medicine syndromes in type 2 diabetes mellitus with chronic heart failure: A clinical study

**DOI:** 10.1097/MD.0000000000021091

**Published:** 2020-07-24

**Authors:** Hui Wang, Jun Zhang, Chun-fang Shi, Jing Jia, Zhi-min Zhang, Jia-jia Sun, Bing-bing Lu

**Affiliations:** a Department of Traditional Chinese Medicine, Majiagou Hospital of Kailuan; b Department of Endocrinology, Tangshan Hospital of Traditional Chinese Medicine; c Department of Internal Medicine, Majiagou Hospital of Kailuan; d Kailuan General Hospital, Tangshan, Hebei, China.

**Keywords:** chronic heart failure, clinical trials, diabetes mellitus, traditional Chinese medicine

## Abstract

**Introduction:**

The incidence of type 2 diabetes has been increasing year by year in recent years. Type 2 diabetes is an important risk factor in the occurrence and development of heart failure, and it is the second potential risk factor after coronary artery disease. At present, there is no unified etiology, pathogenesis, and syndrome differentiation criteria for type 2 diabetes with chronic heart failure, and it is susceptible to subjective factors. Therefore, standardized, objective, and standardized research is needed to provide reference and guidance for clinical diagnosis and treatment. In this study, the theory of syndrome differentiation is used to initially explore the distribution of traditional Chinese medicine syndromes in patients with type 2 diabetes and chronic heart failure through case data collection, syndrome extraction, and clinical data analysis.

**Methods/design:**

In this study, we will collect at least 500 cases of type 2 diabetes with chronic heart failure that meet the standard outpatient and hospitalization, and fill out the case information collection form. Then we will collect a number of clinical diagnosis and treatment information, and judge the syndrome based on the sum of the contribution of each syndrome to the relevant syndrome. We will use Microsoft Excel to establish a database, enter the relevant diagnosis and treatment, and syndrome information of the case information collection table, and verify and correct in time to ensure the accuracy of the data.

**Discussion:**

This study will provide reference and guidance for the clinical diagnosis and treatment of type 2 diabetes with chronic heart failure.

**Trial registration:**

ClinicalTrials.gov, ChiCTR2000033010, Registered on May 18, 2020.

## 1 Introduction

In recent years, the incidence of diabetes mellitus (DM) has increased at an alarming rate. There are about 1.2 million diabetic patients in China every year, and about 90% of them are type 2 DM (T2DM).^[1]^ T2DM is an important risk factor in the occurrence and development of heart failure,^[2,3]^ and it is the second potential risk factor after coronary artery disease.^[4]^ Its associated insulin resistance, hypertension, dyslipidemia, age, obesity, and infection are also risk factors for heart failure.^[5]^ Once heart failure occurs in DM patients, the mortality rate will increase 10 times, and the 5-year survival rate is only 12.4%.^[6]^ In addition, heart failure is also an independent risk factor for T2DM. About 15% to 35% of nondiabetic heart failure patients can develop DM, which leads to an increase in new-onset DM.^[7]^ Clinically, the morbidity and mortality of T2DM combined with chronic heart failure (CHF) are increasing year by year, which seriously affects the survival rate and quality of life of patients. It is a problem worthy of attention in the current clinical frontline. T2DM patients due to coronary artery disease, hypertension, cardiomyopathy, diabetic microangiopathy, abnormal extracellular matrix formation, endothelial dysfunction, renin angiotensin system and sympathetic nervous system activation, insulin resistance and other factors, and complex mechanisms. The synergistic effect of a wide range of comorbid or complicated cardiovascular diseases leads to fibrosis, necrosis, and even apoptosis of cardiomyocytes.^[8]^ Both primary myocardial damage (diabetic cardiomyopathy) and ischemic myocardial damage (combined with coronary heart disease and myocardial ischemia) can promote the occurrence and progression of heart failure, so its incidence and prevalence are both significantly higher than nondiabetics. In addition, heart failure is also an independent risk factor for T2DM.^[7]^ After heart failure occurs, the body activates the sympathetic nervous system and the renin-angiotensin-aldosterone system through the mechanism of neurohumoral regulation, which aggravates insulin resistance and increases blood glucose; increased catecholamine concentration in myocardial tissue causes cardiomyocyte apoptosis; blood vessels. Overexpression of angiotensin II and excessive production of aldosterone can promote myocardial collagen synthesis and accelerate the formation of myocardial fibrosis, which in turn aggravates heart failure and forms a vicious circle.^[9]^ Traditional Chinese medicine (TCM) holds that CHF combined with T2DM is a compound disease syndrome, and its pathogenesis is complicated and intertwined, and the syndrome types are complicated and change. Due to the influence of DM factors, it has its own particularity. Modern medicine has increasingly perfected the understanding of the etiology, pathogenesis, energy metabolism, and other aspects of T2DM combined with CHF, and standardized treatment programs have made great progress. However, there are no special effective measures for the prevention and treatment of the disease, and there are certain limitations that cannot meet the growing health needs of patients. TCM treatment T2DM combined with CHF has flexibility and diversity. Compared with single-component chemical preparations, various components of TCM compound can cooperate with each other to play a therapeutic role. TCM belongs to multitarget, multichannel, and multilink comprehensive treatment. To achieve the purpose of controlling blood sugar, regulating blood lipids, preventing, and treating myocardial fibrosis, and improving cardiac function.

At present, the research on the etiology and pathogenesis, syndrome type, clinical efficacy, etc, of this disease is mostly concentrated on the research level of single disease, and few diseases are involved. Regarding the understanding of the etiology and pathogenesis of CHF, TCM holds that it is a combination of the deficiency and the truth, and it is often combined with phlegm and drinking. Qi deficiency and blood stasis are the basis of heart failure syndrome.^[10]^ Some studies^[11,12]^ have shown that the heart disease is mainly heart, lung, and kidney. T2DM combined with CHF is a complex disease, its pathogenesis and syndrome types are complex and diverse, and the manifestations of syndromes at different stages of the disease are very different. The relevant literature reports on this type of heart failure are very low, and there is a lack of research on syndromes. Clinical diagnosis and treatment of this disease are mostly based on personal experience and simple case summary. Moreover, there is a big difference from the common CHF patients in symptom performance and syndrome differentiation treatment. At present, there is no unified etiology, pathogenesis, and syndrome differentiation criteria for T2DM combined with CHF, and it is susceptible to subjective factors. Therefore, standardized, objective, and standardized research is needed to provide reference and guidance for clinical diagnosis and treatment. The syndrome differentiation system advocated by Professor Zhu Wenfeng^[13]^ is based on the syndrome differentiation theory, which distinguishes syndromes based on syndromes and the combination of syndromes and syndromes to form a syndrome-certification Dialectical thinking process. Syndrome is the pathologic essence determined by the identification of syndromes and is the key to syndrome differentiation. In this study, the theory of syndrome differentiation is used to explore the distribution rules of TCM syndromes in patients with T2DM and CHF through case data collection, syndrome extraction, and clinical data analysis. We will explore the relationship between syndromes and disease influencing factors, summarize the basic pathogenesis of the disease, and provide a certain theoretical basis and scientific guidance for clinically reasonable and standardized treatment. We aim to play a positive role in the standardization, standardization, and internationalization of TCM.

## 2 Methods/design

### 2.1 Study design and settings

**Figure 1:**
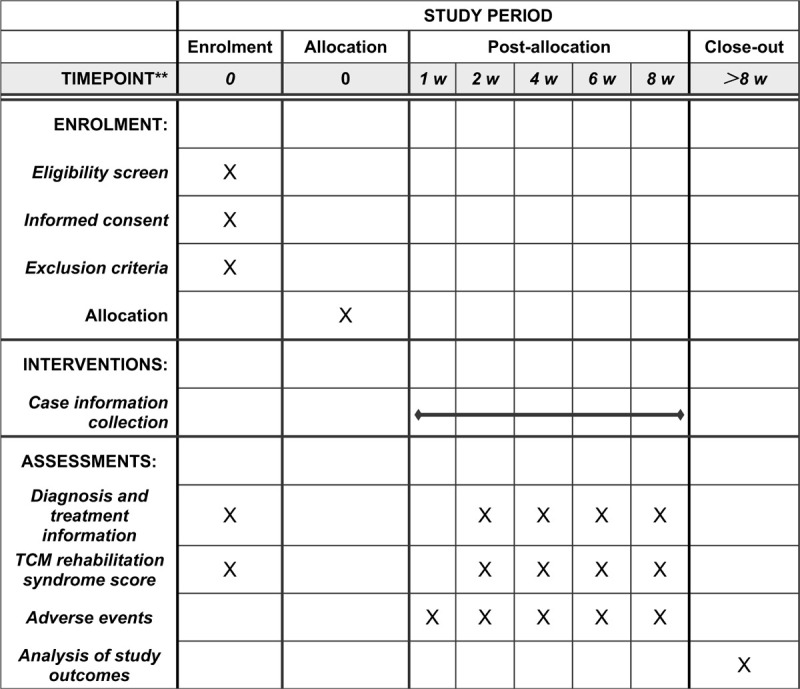
SPIRIT figure for the schedule of enrollment, interventions, and assessments.

This study will be conducted at the Kailuan Majiagou Hospital and Tangshan Hospital of Traditional Chinese medicine (Tangshan City, Hebei Province). This protocol was written and based on Standard Protocol Items: Recommendations for Interventional Trials guidelines. The participants will be informed about the research, procedures, risks, and benefits by BBL (author of this protocol). If they agree, they will sign an informed consent form. Only those participants who read and agree to the protocol and who sign the informed consent form will take part of the study, following the schedule described in Figure 1.

### 2.2 Participants

The subjects of this study will be included in the outpatient and hospitalized patients from the Department of DM Specialist of the Kailuan Majiagou Hospital (Tangshan City, Hebei Province), and meet the diagnostic criteria for T2DM and CHF.

#### 2.2.1 Diagnostic criteria

1. Diagnostic criteria for T2DM. The diagnostic criteria adopted in accordance with the guidelines for the prevention and treatment of T2DM in China (2017 edition): those with typical DM symptoms, such as polyuria, polyphagia, excessive drinking, and unexplained weight loss; plasma glucose level ≥11.1 mmol/L (200 mg/dL); or fasting plasma glucose level ≥7.0 mmol/L (126 mg/dL); or fasting glucose is the critical value, glucose tolerance test, after meals 2 hours blood glucose level ≥11.1 mmol/L (200 mg/dL); exclude type 1 diabetes, special type diabetes, and gestational diabetes.
2. Diagnostic criteria for CHF. We will refer to the diagnostic criteria for Framingham heart failure (Table 1). It mainly includes body weight loss ≥4.5 kg after treatment for more than 5 days. Those who meet 2 major criteria, or 1 major criterion, and 2 minor criteria can be diagnosed with CHF.
3. Classification criteria for cardiac function. We will refer to the NYHA cardiac function grading standard of 1928 (Table 2).

**Table 1:**
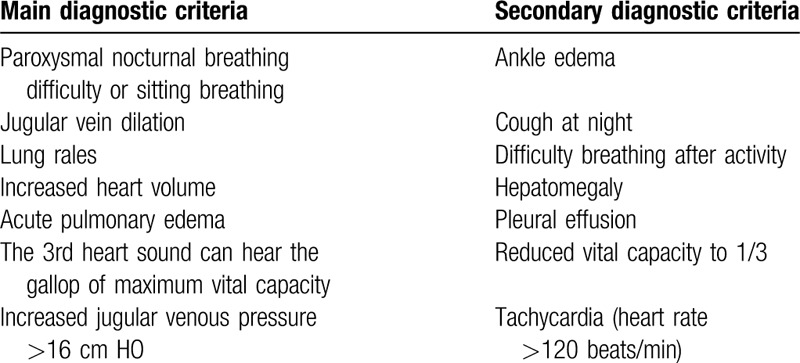
Diagnostic criteria of Framingham heart failure.

**Table 2:**
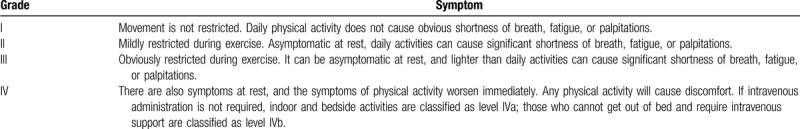
NYHA cardiac function grading standards.

#### 2.2.2 Inclusion criteria

This study will be conducted in China. Patients will be recruited from Plastic Surgery Departments of the Kailuan Majiagou Hospital. We will enroll participants based on the following inclusion criteria:

1. Comply with the diagnostic standards of western medicine T2DM and CHF.
2. Cardiac function grading (grading) belongs to 2 to 4 patients.
3. The age is between 45 and 85 years old.
4. Those who have voluntarily signed the informed consent;

#### 2.2.3 Exclusion criteria

Patients will be excluded if they meet the following criteria:

1. Patients with severe diseases such as malignant hypertension, severe arrhythmia, acute myocardial infarction, cerebrovascular accident, etc.
2. Patients with acute infection, trauma, surgery, diabetic ketoacidosis, and hyperosmolar coma recently.
3. Patients with severe primary diseases such as brain, liver, kidney and hematopoietic system, mental illness, and cognitive dysfunction.
4. Patients with tumor and other organ failure.

#### 2.2.4 Conditions for participants to suspend and withdraw from the clinical trial

Researchers participating in clinical trials should carefully record the reasons for the suspension of the trial and the relationship with the clinical trial. It is necessary to clearly record the unwillingness of the subjects to continue the clinical trials, put forward the reasons for withdrawing from the clinical trials, and record the evaluation indicators at the time of discontinuation in detail.

1. The main symptoms are not clear; the extended description of the current medical history is incomplete; the chief complaint or the description of the current medical history does not match the diagnosis of this disease.
2. Incomplete records of tongue coating and pulse of TCM affect the syndrome judgment of this disease.
3. The lack of or incomplete general information and physical and chemical index information affects the analysis of relevant data.

### 2.3 Interventions

Case information collection: We will collect inpatient records and outpatient cases of patients with T2DM and CHF admitted to Majiagou Hospital in Kailuan. We will fill in the case information collection form to collect a variety of clinical diagnosis and treatment information:

1. Basic information: medical record number, admission time, name, gender, age, etc.
2. Brief medical history: symptoms, signs, tongue, pulse, medical history, course of T2DM, physical examination, diagnosis, etc.
3. Auxiliary examination: including glycosylated hemoglobin (HbAlc), triglyceride, cholesterol, urine microalbumin, *N*-terminal pronatriuretic peptide (NTProBNP), left ventricular ejection fraction, liver and kidney function.

### 2.4 Statistical analysis

1. Collect clinical diagnosis and treatment information (such as symptoms, signs, electrocardiogram, tongue, TCM pulse and laboratory examination data, physical examination data, etc), establish a database, verify every 10 medical records entered, and correct in time to ensure data accuracy.
2. The data is analyzed with the help of SPSS 25.0 statistical software. The measurement data are expressed by *x* ± *s* or *M*, the count data are expressed by the composition ratio or rate, and the Chi-squared test is used for the comparison between the groups. The model analyzes the relationship between relevant syndromes and clinical indicators.

### 2.5 Data management

Data management uses EXCEL software to build a database, double entry, check for outstanding values, and lock. Information obtained from the evaluation of each participant will be recorded on a paper print-out. The information will then be handwritten on a paper document case report form and entered into an Excel file for future statistical analyses. In accordance with the Personal Information Protection Act, the names of all participants will not be disclosed, and a unique identifier number given during the trial will be used to identify participants. All of the participants will be informed that the clinical data obtained in the trial will be stored in a computer and will be handled with confidentiality. The participants’ written consent will be stored by the principal investigator.

### 2.6 Ethics

The study will be conducted under the Declaration of Helsinki principles, as well as following the norms of good clinical practice. Recruitment of patients has not started in this study. The study plan will be submitted to the ethics committee of the Tangshan Hospital of Traditional Chinese medicine for review. The study protocol will be approved by the ethics committee of Tangshan Hospital of Traditional Chinese medicine. The protocol of this study has been registered in the Chinese Clinical Trial Registry with the number ChiCTR2000033010.

## 3 Discussion

With the development of economy, improvement of living standards, changes in lifestyle, changes in diet structure, and the aging of the population, the incidence of DM is increasing at an alarming rate.^[14]^ In 2017, the DM International Alliance announced that the number of diabetics worldwide reached 42.5 billion, with a prevalence rate of about 88%, an increase of nearly twice the 151 million in 2000. China ranks first in the world with 114.4 million patients, with a prevalence rate of 10.9%, an increase of about 1.2 million diabetic patients each year, of which about 90% are T2DM patients. The prevalence of DM increases significantly with age, men are significantly higher than women, cities are significantly higher than rural areas, about twice as high in rural areas, but the mortality rate of DM patients in rural areas is higher than that in cities.^[15,16]^ T2DM is an important risk factor in the occurrence and development of heart failure, and it is the 2nd potential risk factor after coronary artery disease. In 2004, the annual meeting of the European Association for Diabetes Research (EASD) pointed out that when T2DM occurs, patients have grade I heart failure. DM with heart failure is the main outcome of patients with T2DM.^[17]^ The prevalence of heart failure in diabetic patients is 2.5 to 3 times higher than that in the general population.^[18]^ About 30% of patients with heart failure and 25% of patients with drug-refractory heart failure have DM, and the proportion of diabetic patients with heart failure increases year by year Bright. CHF is one of the most common causes of death in patients with DM.^[19]^ Once heart failure occurs in diabetic patients, the mortality rate will increase 10 times, and the 5-year survival rate is only 12.4%. In addition, heart failure is also an independent risk factor for T2DM. About 15% to 35% of nondiabetic heart failure patients can develop DM, which leads to an increase in new DM.

The pathogenesis of heart failure has 2 main aspects. On the one hand is primary myocardial damage: long-term glucose and lipid metabolism disorders affect the metabolism of myocardium, vascular endothelium, and other tissues, promoting cardiac microvascular endothelial cell proliferation, thickening of the basement membrane, reduced oxygen utilization, myocardial fibers and perivascular fibrosis, myocardial glycoproteins, collagen fibers, triglycerides, and cholesterol deposits.^[20]^ These abnormal metabolisms lead to myocardial energy metabolism disorders and decreased energy reserves, which damages myocardial cells and reduces excitability, resulting in left ventricular diastolic dysfunction. With diabetic cardiomyopathy the most common.^[21]^ The other is ischemic myocardial damage: coronary atherosclerotic stenosis, myocardial remodeling after myocardial infarction, myocardial pathologic changes, stenosis and ischemic necrosis caused by atherosclerotic plaque, myocardial interstitial fibrosis necrosis, inflammation cell infiltration, marked thickening of the inner lining of the small blood vessel wall, a large amount of glycosylated acetabular deposits, and changes in nerve fibers, local spindle and spherical thickening of nerve fibers, etc, lead to weakened ventricular muscle function, decreased filling, and myocardial hypertrophy, ventricular enlargement, ventricular remodeling, and heart failure.^[22]^ Coronary heart disease with myocardial ischemia and/or myocardial infarction is the most common cause of heart failure. T2DM patients can induce heart failure from both aspects of the above pathogenesis, so their incidence and prevalence are significantly higher than those of nondiabetics. In addition, heart failure is also an independent risk factor forT2DM.^[23]^ After heart failure occurs, the body activates the sympathetic nervous system and the renin-angiotensin-aldosterone system through the mechanism of neurohumoral regulation, which aggravates insulin resistance and increases blood glucose; the increase in the concentration of catecholamines in the myocardial tissue causes the myocardial cells to wither death; the overexpression of angiotensin II and the excessive production of aldosterone can promote the formation of myocardial collagen and accelerate the formation of myocardial fibrosis, thereby exacerbating heart failure and forming a vicious circle.

The TCM holds that CHF combined with T2DM is a compound disease syndrome, and its pathogenesis is complicated and intertwined, and the syndrome types are complex and diverse.^[24]^ Due to the influence of DM factors, it has its own particularity. Modern medicine has increasingly perfected the understanding of the etiology, pathogenesis, energy metabolism, and other aspects of T2DM combined with CHF, and standardized treatment programs have made great progress. However, there are no special effective measures for the prevention and treatment of the disease, and there are certain limitations that cannot meet the growing health needs of patients.^[25]^ TCM treatment T2DM combined with CHF has flexibility and diversity. Compared with single-component chemical preparations, various components of TCM compound can cooperate with each other to play a therapeutic role. TCM belongs to multitarget, multichannel, and multilink comprehensive treatment.^[26]^ To achieve the purpose of controlling blood sugar, regulating blood lipids, preventing and treating myocardial fibrosis, and improving cardiac function. At present, there is no unified etiology, pathogenesis, and syndrome differentiation criteria for T2DM combined with CHF, and it is susceptible to subjective factors. Therefore, standardized, objective, and standardized research is needed to provide reference and guidance for clinical diagnosis and treatment. In this clinical study, we will screen no fewer than 500 cases that meet the inclusion criteria, collect clinical diagnosis, and treatment information, apply syndrome differentiation theory, extract syndromes, and establish a database. Then we will enter the relevant clinical indicators and syndrome information, statistical analysis of the data. This study will initially explore the distribution of TCM syndromes in T2DM patients with CHF and summarize the basic TCM pathogenesis of this disease.

## Acknowledgments

The authors thank all the trial participants. The authors are grateful for the support for this study: trial coordinating team, surgical staff, nurses, and research departments.

## Author contributions

HW, JZ, PPH, and CFS designed the study protocol and drafted the manuscript. JJ and BBL reviewed the study protocol and drafted the manuscript. ZMZ and JJS are responsible for the statistical design and analysis as trial statistician. All authors carefully read and approved the final version of the manuscript.
